# Talimogene Laherparepvec (T-VEC): A Review of the Recent Advances in Cancer Therapy

**DOI:** 10.3390/jcm12031098

**Published:** 2023-01-31

**Authors:** Tiantian Zhang, Tony Hong-Ting Jou, Jerline Hsin, Zhe Wang, Kelly Huang, Jian Ye, Holly Yin, Yan Xing

**Affiliations:** 1 Toni Stephenson Lymphoma Center, Department of Hematology and Hematopoietic Stem Cell Transplantation, Beckman Research Institute, City of Hope, Duarte, CA 91010, USA; 2 School of Medicine, National Yang Ming Chiao Tung University, Taipei 11217, Taiwan; 3 Department of Pharmacy, City of Hope, Duarte, CA 91010, USA; 4 High Throughput Screening Core, Department of Share Resources, Beckman Research Institute, City of Hope, Duarte, CA 91010, USA; 5 Department of Medical Oncology and Therapeutics Research, City of Hope, Duarte, CA 91010, USA; 6 Department of Immuno-Oncology, Beckman Research Institute, City of Hope, Duarte, CA 91010, USA

**Keywords:** oncolytic virotherapy, T-VEC, immune checkpoint inhibitors, immunotherapy, targeted therapy, combinational therapy, melanoma, cutaneous cancers, clinical trials

## Abstract

The landscape of melanoma treatment has undergone a dramatic revolution in the past decade. The use of oncolytic viruses (OVs) represents a novel therapeutic approach that can selectively infect and lyse tumor cells and induce local and systemic antitumor immune responses. As the first OV approved by the Food and Drug Administration (FDA) for melanoma treatment, talimogene laherparepvec (T-VEC), a genetically modified herpes simplex virus (HSV), has shown promising therapeutic effects in the treatment of advanced melanoma, both as a monotherapy or in combination with other immunotherapies, such as the immune checkpoint inhibitors (ICIs). With proven efficacy, T-VEC has been evaluated against a variety of other cancer types in a clinical trial setting. In this article, we will provide a review on OVs and the application of T-VEC in melanoma monotherapy and combination therapy. In addition, we will review the recent progress of T-VEC application in other cutaneous cancer types. Moreover, we will briefly describe our experience of T-VEC therapy at City of Hope, aiming to provide more insight for expanding its future application.

## 1. Introduction 

The last decade has witnessed a dramatic transformation of the landscape of melanoma treatment. Based on the deeper understanding of the molecular features of melanoma and the tumor microenvironment, the current melanoma therapies have progressed to mainly include targeted therapy, immune checkpoint inhibitors (ICIs), and virotherapy. The elucidation of BRAF V600 mutations and the dysregulated RAS/RAF/MEK/ERK pathway in melanoma cells has led to the development of targeted therapies including BRAF inhibitors (BRAFi) and MEK inhibitors (MEKi), which have shown significant efficacy in melanoma eradication and been approved by the Food and Drug Administration (FDA). The discovery of ICIs mainly includes the antibodies against cytotoxic T lymphocyte antigen-4 (CTLA-4), programmed cell death protein-1 (PD-1), and programmed death-ligand 1 (PDL-1). ICIs have provided another approach by releasing the inhibitory brakes on the T cells and facilitating robust immune responses, rendering them effective in melanoma treatment [1,2]. 

Oncolytic viruses (OVs) represent a novel class of cancer therapy in which wild-type or genetically modified viruses are used. Historically, viruses have been explored as therapeutics in two ways—as viral vectors for gene therapy and tumor-lysing (“oncolytic”) viruses [3]. The key difference between these two categories lies in that the OVs are typically replication-competent, whereas the viral vectors for gene therapy are usually replication-defective viruses. Interestingly, modern OVs have often been engineered to express immunostimulatory proteins, which also fulfill the function as viral vectors. A variety of viruses, such as herpes simplex virus (HSV), vaccinia virus, adenovirus, and reovirus, have been evaluated for their oncolytic potency. While some of these viruses have completed different phases of clinical trials, talimogene laherparepvec (T-VEC), which is an engineered HSV-1 with the insertion of the granulocyte monocyte colony-stimulating factor (GM-CSF) gene and deletion of infected cell protein 34.5 (ICP34.5) and ICP47 genes, is the first OV approved by the FDA for melanoma treatment [4,5]. In this review, we will focus on T-VEC and its effects on melanoma and other cutaneous malignancies as a monotherapy and in combination with other cancer therapies (Table 1). We will also discuss ongoing trials involving T-VEC (Table 2). Moreover, we will look at how City of Hope Comprehensive Cancer Center provides T-VEC treatment to its patients, which will provide insight into the implementation of T-VEC in the real-world. In this review, the novelty lies in (1) providing an overview of the path the T-VEC took from initial testing to widespread use, (2) offering detailed information on the past and ongoing clinical trials involving the use of T-VEC as a monotherapy and in combination therapy, (3) and presenting a general description of the clinical experience with T-VEC at City of Hope.

## 2. Overview of Oncolytic Virus and T-VEC

OVs have emerged as a novel class of immunotherapies with remarkable efficacy through possessing two closely related properties: the capability to kill cancer cells and the potential to enhance anti-tumor immune responses [17]. The viruses, either native or modified, are able to infect and replicate within tumor cells, causing cell lysis and the release of viral progenies that will proceed to infect neighboring cells. Moreover, virus infection is able to trigger an apoptosis cascade in the surrounding cancer cells, which limits the viral replication and tumor cell proliferation. Meanwhile, the rupture of the tumor cells releases tumor-derived antigens that are new to the immune system, thereby facilitating the development of systemic tumor-specific immune responses [17]. 

In comparison to normal cells, which possess intact antiviral mechanisms, tumor cells have been found to have abnormally regulated pathways that can be manipulated to facilitate OV infection and replication. For instance, melanoma cells have been shown to harbor Ras overexpression and defective interferon (IFN)-signaling pathways, which can be readily targeted by the oncolytic vesicular stomatitis virus (VSV) and reovirus [18]. Additionally, while tumor cells often overexpress tyrosinase and survivin, the genetic modification of the viral genome to incorporate the promoters of tyrosinase or survivin genes has been found to increase the oncospecificity of oncolytic viruses. Moreover, to stimulate tumor-specific immune reactions, OVs have been genetically engineered to express an array of immunomodulatory or immunostimulatory proteins, such as interleukin (IL)-2, IFNγ, and GM-CSF [17].

In the past two decades, a wide variety of viruses, including adenovirus, HSV, and poxvirus, have been studied for their potency as oncolytic viruses [19,20,21]. T-VEC, an attenuated HSV expressing GM-CSF, became the first oncolytic agent that achieved regulatory approval in the United States, Europe, and Australia. As a JS1 strain of HSV-1, the preferential tumor infection and replication of T-VEC is enhanced via the deletion of the ICP34.5 gene, which also attenuates the natural neurovirulence of the virus and improves the safety [22]. The insertion of two copies of human GM-CSF gene in the genome of T-VEC leads to local expression, which enhances the recruitment of antigen-presenting cells (APCs). The activation of APCs facilitates the tumor antigen presentation to tumor-specific T cells, which further elevates the antitumor immunity [23]. Another key modification is the deletion of the ICP47 gene. While ICP47 normally reduces antigen presentation by binding to the transport-associated protein to prevent the antigen loading of MHC-I molecules, the deletion of the ICP47 gene enhances tumor antigen presentation. Additionally, the deletion of ICP47 permits the earlier and increased expression of the herpes unique short 11 (US 11) gene, leading to increased selectivity for tumor cells [24].

## 3. T-VEC Treatment for Melanoma 

### 3.1. T-VEC Monotherapy for Melanoma and Path to FDA Approval

T-VEC was first tested in a phase I clinical trial published by Hu et al. in 2006, in which T-VEC was administered via intratumoral injection in patients with a wide diversity of tumor types, including refractory breast, head and neck, and gastrointestinal cancers and malignant melanoma. In total, thirty patients were segregated into either a single-dose group, where doses of 10^6^, 10^7^, and 10^8^ plaque-forming units (pfu)/mL were tested, or into a multidose group, which tested a number of dose regimens. While 26 of the enrolled 30 patients were evaluable, 19 of the 26 posttreatment biopsies showed residual tumors, of which 14 exhibited extensive necrosis and apoptosis, and all demonstrated strong staining for HSV in the necrotic areas. A mild toxicity profile was reported, which mainly comprised low-grade fever, chills, myalgia, and local reactions. The dose regimen that consisted of an initial dose of 10^6^ pfu/mL followed by 2 doses of 10^8^ pfu/mL every two to three weeks was reported to be the most effective approach in both seropositive and seronegative patients [6].

In the following phase II clinical trial published by Senzer at al. in 2009, T-VEC (4 mL of 10^6^ pfu/mL followed by 4 mL of 10^8^ pfu/mL every 2 to 3 weeks for up to 24 treatments) was tested in fifty patients with stage IIIc unresectable metastatic melanomas. A mild toxicity profile, including transient flu-like symptoms, was reported. The overall response rate (ORR) per the Response Evaluation Criteria in Solid Tumors (RECIST) was 26%; the complete response (CR) rate was 16% and the partial response (PR) rate was 10%. The regression of both injected and distant lesions was observed, with 92% of the responses being maintained for nearly three years. The overall survival (OS) rates were 58% at 1 year and 52% at 2 years [7].

In the subsequent phase III OPTIM study, intralesional T-VEC was compared with subcutaneous GM-CSF when treating 436 patients with unresected stage IIIB to IV melanomas. While the primary end point was a durable response rate (DRR), which represents an objective response lasting continuously for 6 months per independent assessment, the secondary end points included the OS and ORR. In regard to the T-VEC injection, the first dose was given at 10^6^ pfu/mL (to seroconvert HSV-seronegative patients). Subsequent T-VEC doses of 10^8^ pfu/mL were administered three weeks after the first dose and then once every 2 weeks. GM-CSF 125 µg/m^2^ was administered subcutaneously once daily for 14 days in 28-day cycles [8]. In the final report of this study in 2019, a significantly higher DRR was reported with T-VEC (19.3%) than GM-CSF (1.4%). Similarly, the ORR was greater in the T-VEC (31.5%) than GM-CSF (6.4%) treatment. Fifty patients (16.9%) and one (0.7%) patient in the T-VEC and GM-CSF arms, respectively, achieved CR. The median OS in the T-VEC arm reached 23.3 months (95% CI, 19.5–29.6) versus 18.9 months with GM-CSF (95% CI, 16.0–23.7). The toxicity profile was acceptable, with the most common adverse events (AEs) including fatigue, chills, pyrexia, nausea, and influenza-like illness. While the incidence of these AEs was highest during the first three cycles, most AEs lasted 2–4 days and subsequently subsided over time [9]. Based on the data from the OPTIM study, T-VEC was officially approved by the FDA on 27 October 2015. 

Furthermore, other clinical trials of T-VEC monotherapy have been conducted and have shown promising results in terms of their efficacy and safety. For example, a phase 1 study (NCT03064763) assessed the safety and effectiveness of T-VEC in Japanese patients with advanced stage melanomas that could not be surgically removed. The study found that T-VEC had a favorable safety profile, with no dose-limiting toxicities being observed, and the most common side effects were fever and chills. Most AEs were grade 1 or 2, which were consistent with those observed in the OPTIM trial [25].

### 3.2. T-VEC Combinational Therapy for Melanoma

#### 3.2.1. Rationale for T-VEC Combinational Therapy 

The current frontline therapies for melanoma include chemotherapy, targeted therapy, immune checkpoint inhibitors (ICIs), and virotherapy (i.e., T-VEC). The activating mutation of BRAF, the key serine threonine protein kinase in the RAS/RAF/MEK/ERK pathway, has been found in nearly 70% of melanomas, with the consequential activation of the downstream MEK and ERK signaling contributing to the dysregulated proliferation of melanoma cell growth [26]. Vemurafenib was the first BRAFi approved by the FDA for the treatment of BRAF V600 mutant melanoma, followed by dabrafenib and encorafenib. While the BRAFis all exhibited improved survival outcomes in melanoma patients compared to the traditional chemotherapies, the rapid development of drug resistance to the BRAFi monotherapy was reported. The combination therapy of BRAFi and MEKi was developed subsequently to reduce this resistance, which was proven to be remarkably effective in an array of clinical trials. For instance, in the coBRIM trial, the combination of vemurafenib and cobimetinib resulted in a remarkably improved median OS (22.3 months) and progression-free survival (PFS) (12.3 months) compared to that of the vemurafenib monotherapy (OS, 17.4 months; PFS, 7.2 months) [27]. Similarly, in the COMBI-d trial, treatment with a combinational therapy of trametinib and dabrafenib led to a significantly prolonged median OS (25.1 months vs. 18.7 months) and increased median PFS (11.0 months vs. 8.8 months) in comparison to the dabrafenib monotherapy [28].

Interactions between immune checkpoints and their ligands negatively influence T cell function and the subsequent immune responses against tumor antigens. ICIs, which block these immunosuppressive pathways, have been shown to effectively elevate the antitumor immune reactions in preclinical studies. Among the ICIs, the blockade of CTLA-4 and interaction between PD-1 and PD-L1 are the two most prominent. The development of monoclonal antibodies against CTLA-4 (e.g., ipilimumab) and PD-1 (e.g., nivolumab and pembrolizumab), along with the successful survival outcomes in clinical trials with advanced melanoma patients, has significantly transformed the melanoma treatment landscape. For instance, in the CheckMate067 trial, untreated unresectable stage III or stage IV patients were randomly segregated into ipilimumab, nivolumab, and nivolumab + ipilimumab treatment groups. With a 6.5-year follow-up period, remarkable improvements were reported in the median OS values (19.9 months with ipilimumab, 36.9 months with nivolumab, and 72.1 months with nivolumab + ipilimumab) and median treatment-free intervals (1.9 months, 2.3 months, and 27.6 months with ipilimumab, nivolumab, and nivolumab + ipilimumab, respectively). In addition, 43%, 74%, and 81% of the patients after ipilimumab, nivolumab, and nivolumab + ipilimumab treatment, respectively, received no further subsequent systemic therapy [29,30]. 

While T-VEC, ICIs, and targeted therapies exhibit remarkable success, the combination of T-VEC with ICIs or targeted therapies would be expected to have synergistic efficacy. It has been shown that T-VEC infection and replication in tumor cells can elevate the inflammatory state of the tumor microenvironment, which can further promote T cell influx and activation [31]. While the GM-CSF gene product facilitates the recruitment and activation of antigen presentation cells (APCs), the oncolysis of the tumor cells spreads the tumor-associated antigens, which increases the availability to APCs and T cell priming. As the immune responses can be reduced via the expression of immune checkpoints on the T cells, such as CTLA-4 and PD-1, the coadministration of ICIs can prevent T cell exhaustion and prolong T cell activation and expansion [32]. 

#### 3.2.2. Clinical Trials of T-VEC Combinational Therapy for Melanoma

The first randomized trial assessing the efficacy of the combinational therapy of T-VEC and ICIs was reported by Chesney et al. One hundred and ninety-eight patients with unresectable stage IIIB to IV melanomas were randomly segregated into the T-VEC + ipilimumab (n = 98) or ipilimumab monotherapy (n = 100) group. The toxicity profile was reported as mild, and the AEs mainly included fatigue, chills, and diarrhea. While three patients in the combination therapy group had fatal AEs, none were related to the treatment itself. The objective response was reported as thirty-eight patients (39%) in the combination therapy group and 18 patients (18%) in the ipilimumab monotherapy group. The median time to response was 5.8 months in the T-VEC + ipilimumab group (n = 38), which was not estimable in the ipilimumab group (n = 18). The median PFS was 8.2 months in the duplet group and 6.4 months in the monotherapy group. While this study indicates that the combination has greater antitumor activity without additional safety concerns compared to ipilimumab, several interesting findings are noted. First, it was notable that both the injected lesion and visceral lesions decreased in size in response to treatment. In total, 52% of the patients receiving combination therapy and 23% of the patients receiving ipilimumab monotherapy had visceral lesions that responded to treatment. Second, the efficacy of the treatments was shown to be affected by the tumor staging and existence of BRAF mutations. The ORR in the combination therapy group was significantly higher for patients with low tumor staging (IIIB/IIIC/IVM1a) in comparison to high tumor staging (IVM1b and IVM1c) (44% vs. 33%). The ORR in the combination arm was 42% among BRAF wild-type patients, which was greater than that among BRAF mutation patients (34%) [10]. 

In the other trial, the MASTERKEY-265 trial (phase Ib/III study), T-VEC + pembrolizumab was evaluated versus pembrolizumab monotherapy. In the phase Ib study, 21 patients with unresectable stage IIIB-IVM1c melanoma with injectable, measurable lesions and no prior systemic treatment were enrolled and followed for 18.6 (17.7–20.8) months before the time of reporting. There were no severe toxicities reported in any of the 21 patients, with the most common AEs including fatigue, chills, and fever. With the combinational therapy, the confirmed objective response rate was 61.9% (95% CI, 38.4–81.9%), while the confirmed CR rate was 33.3% (95% CI, 14.6–57.0%). Moreover, the combination treatment led to >50% reductions in 82% of injected, 43% of non-injected non-visceral, and 33% of non-injected visceral lesions [11]. All twenty-one patients enrolled were off treatment as of the data cutoff (Mar 2, 2020). Among them, 6 died and 15 are in long-term follow-up. With a median follow-up time of 58.6 months, the CR rate was reported as 43% (9/21 patients); 92.3% of the responders (12/13) remained in response, including all 9 patients with a CR. While the median PFS and OS were not reached at the data cutoff point, the 4-year PFS and OS rates were estimated as 55.9% and 71.4%, respectively. No additional safety signals were ever detected [33]. 

The remarkable results of the phase 1b part of MASTERKEY-265 led to the phase III randomized, double-blind KEYNOTE-034 study. In this study, a total of 692 patients with unresectable stage III-IVM1c melanoma who were naive to anti-PD1 therapy were randomized 1:1 to a T-VEC + pembrolizumab or placebo + pembrolizumab treatment. With a median follow-up of 31.0 months, it was reported that the median PFS was 14.3 months for the T-VEC + pembrolizumab arm and 8.5 months for the placebo + pembrolizumab arm. While the median OS was not reached for the T-VEC + pembrolizumab arm, the OS of the placebo + pembrolizumab arm was 49.2 months. However, statistical significance was not expected with OS in the primary OS analysis. The ORRs were 48.6% for the T-VEC + pembrolizumab group and 41.3% for the placebo + pembrolizumab group. The CR rate was greater in the T-VEC + pembrolizumab arm in comparison to the placebo + pembrolizumab arm (17.9% vs. 11.6%). The DRRs were 42.2% in the T-VEC + pembrolizumab arm and 34.1% for the placebo + pembrolizumab arm. Importantly, the safety profiles were acceptable, without any unknown safety issues from each agent [12].

In addition to the abovementioned trials, several other clinical trials involving the T-VEC combination therapy are ongoing to further evaluate the systemic efficacy of T-VEC. For instance, in a phase II clinical trial (NCT#02965716), patients with unresectable stage IIIB-IV melanoma who did not respond to PD-1/PD-L1 blockade were treated with T-VEC + pembrolizumab. This study had been designed to evaluate the T cell infiltration into tumors, the T-cell receptor (TCR) clonality in tumors and in peripheral blood, and the tumor immune microenvironment after T-VEC + pembrolizumab combination treatment, which will hopefully provide more in-depth information on the mechanisms of T-VEC in tumor eradication [34]. 

## 4. T-VEC Treatment in Other Cutaneous Cancer Types

Along with the success of T-VEC in melanoma treatment, T-VEC monotherapy and combination therapies are under exploration in other cutaneous cancer types, such as Merkel cell carcinoma (MCC) and cutaneous squamous cell carcinoma (CSCC).

As an aggressive malignancy from cutaneous neuroendocrine cells, MCC typically presents on the sun-exposed areas in the elderly. The current FDA-approved treatment for MCC includes chemotherapy and ICIs, such as PD-1 or PD-L1 blockade. Recent clinical trials reported superior ORR and PFS values with PD-1/PD-L1 treatment in comparison to chemotherapy; however, the CR rate was low, and most patients progressed in less than 12 months [35]. In regard to these observations, T-VEC has been assessed for MCC therapy. In Westbrook et al., four patients with regionally advanced MCC were treated with T-VEC. All four patients achieved durable CRs, with a median PFS of more than 16 months without severe AEs. Moreover, the treatment with T-VEC prevented distant metastasis in these high-risk individuals [14]. In another study, Knackstedt et al. reported on the combination therapy of T-VEC and a PD-1/PD-L1 inhibitor in two patients with anti-PD-1 refractory MCC. While the radiotherapy and chemotherapy had been utilized with failure, the T-VEC and PD-1/PD-L1 inhibitor combination therapy led to CR in one patient and near-CR in another patient [15]. 

CSCC is another common cutaneous malignancy, which has a wide range of presentations from low-risk in situ disease to high-risk advanced metastatic tumors. Compared to melanoma, CSCC has a less aggressive clinical course but a significantly higher incidence rate [36]. The current treatment options mainly include PD-L1 inhibitors, chemotherapy, and EGFR inhibitors. A single-arm phase II trial of T-VEC (NCT03714828) was conducted in treating low-risk invasive CSCC. With the Simon 2-stage design being used and a total sample size of 20 patients, 7 patients were recruited for stage 1 and an additional 13 patients would be recruited if five or more subjects met the primary endpoint in stage 1. In the interim analysis of 7 patients, all achieved overall CR. All AEs were of grades 1–2 based on the NCI Common Terminology Criteria for Adverse Events v. 4.0 (CTCAE v. 4.0), with the most common AEs including transient fatigue, flu-like symptoms, and headaches. At the time of analysis, the mean time to response was 43.4 days and the duration of the ORR was 190 days [37]. While-T-VEC has shown remarkable success with a 100% CR in stage 1, a high response rate will be expected and assessed at the completion of the study.

Currently, several other clinical trials are ongoing for assessing the efficacy of T-VEC in treating these cutaneous malignancies. For instance, the combination of T-VEC and radiotherapy is being evaluated in MCC and melanoma in a phase II trial (NCT02819843) [16]. In another phase II trial (NCT02978625), a combination therapy of T-VEC and nivolumab is being assessed in MCC, CSCC, and basal cell carcinoma [38,39,40,41]. 

## 5. T-VEC Treatment Practices in City of Hope

**Figure 1:**
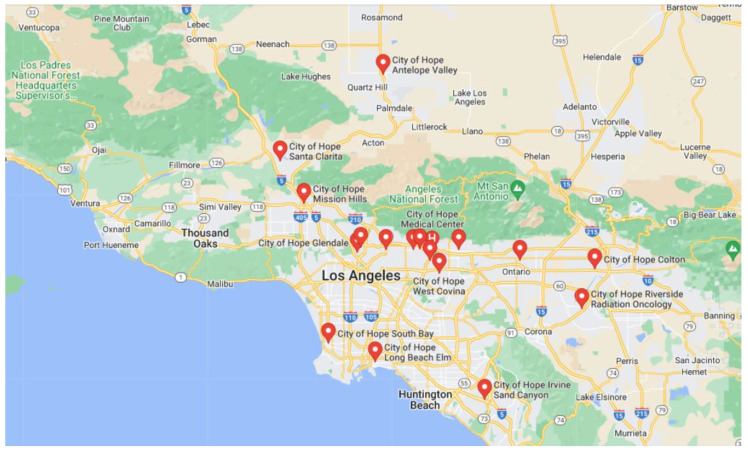
Map of City of Hope locations in Southern California. Teardrop with “H” represents COH main campus at Duarte, California, where the T-VEC treatment is performed. Other red teardrops represent 18 out of 27 campuses in City of Hope.

City of Hope is a National Cancer Institute (NCI)-designated Comprehensive Cancer Center and a member of the National Comprehensive Cancer Network (NCCN) (Figure 1). At City of Hope, T-VEC treatment has been applied to patients with recurrent or metastatic melanoma, metastatic CSCC, and metastatic MCC. While a few patients complained of chills, fever, and fatigue a few hours after T-VEC injection and some edema at the injection site, these symptoms usually lasted less than 24 h. Extensive fibrosis has been observed after T-VEC injection, which prevented further intratumoral injections. Overall, the toxicity profile of T-VEC has been reported as mild and tolerable. 

Among the melanoma patients under T-VEC treatment, nearly 32% of the patients were referred from other hospitals for either monotherapy or combination therapy. Overall, in comparison to T-VEC monotherapy, T-VEC + ICI combination therapies in which pembrolizumab was applied most frequently have resulted in higher CR rates, which indicates synergistically the more significant efficacy with the addition of ICIs. In light of this observation, we are currently undertaking preclinical studies that aim to explore melanoma treatment with the intratumoral injection of multi-drug combination therapies. Regarding the subsequent therapies following T-VEC, the PD-1 or CTLA-4 inhibitors as monotherapies or in combination and the BRAF or MEK inhibitors as monotherapies or in combination were most commonly administered. A small number of patients with metastatic CSCC and MCC were treated with either T-VEC monotherapy or combination therapies as off label treatments per the tumor board recommendations. While most patients with metastatic CSCC and MCC suffered from the progression of disease before eventually expiring, future trials on CSCC and MCC patients need to be conducted before the efficacy of T-VEC can be fully assessed in these two malignancies.

While most of the patients who were referred to City of Hope for T-VEC treatment lived within reasonable distance (less than 50 miles from City of Hope), several resided far away and even travelled four to five hours one way to receive treatment. Meanwhile, the regulations for the transportation, storage, and handling of T-VEC are cumbersome. For instance, T-VEC is usually stored frozen at −70 to −90 °C then thawed to a liquid state prior to preparation, which takes approximately 30 to 70 min in our experience. The pharmacy workflow must be adjusted so that trained technicians can prepare the syringes and the IV hood must be set aside for cleaning to reset the airflow. The main constraints include the lack of trained providers who can administer T-VEC, the freezer availability and capacity, and the biweekly scheduling. Additionally, insurance may not approve T-VEC for indications other than melanoma. All of these factors have limited the access of patients to T-VEC treatment. 

## 6. Discussion and Future Directions

The landscape for cancer treatment has been rapidly evolving in the last few decades. With the advent of new drugs and combinations, the therapeutic options for patients have widely broadened and become more multidisciplinary. As the first OV approved by the FDA, T-VEC provides a new approach for cancer therapy regimens. 

T-VEC was first studied in clinical trials of melanoma and demonstrated improved efficacy. For instance, in the phase III OPTIM study involving patients of unresected stages IIIB to IV melanoma, significantly higher ORR, DRR, and CR values with tolerable toxicity profiles were associated with the treatment of T-VEC in comparison to GM-CSF. With FDA approval, its application has been rapidly extended to the treatment of other cancer types. For instance, in a single-arm phase II trial where T-VEC was administered in patients with invasive CSCC, all 7 patients in stage 1 of the study achieved CR, with very mild AEs. Currently, T-VEC alone or as part of a combination therapy has been explored in clinical trials with a variety of cancers, such as MCC, CSCC, breast cancer, pancreatic cancer, colorectal cancer, and liver cancer. However, it is noteworthy that so far in most of the clinical trials, intratumoral injection remains the only option for virus administration. In fact, the intratumoral administration of T-VEC causes the direct lysis of tumor cells and increases the intratumoral infiltration of APCs and T cells, which leads to neoantigen recognition and strengthened tumor specificity. Moreover, intratumor injection protects the virus from the neutralizing antibodies and macrophage sequestration effect towards the virus. While the intratumoral route serves as a perfect means of eradication of locoregional cancers, it might not be effective with distant tumors that are inaccessible to direct injections or metastasized tumors that cannot be accurately located. Clinical trials are ongoing to evaluate the systemic route of OVs, which have demonstrated feasibility in systemic injections. More results are still needed to show the antitumor efficacy that can be achieved. 

While ICIs have been commonly used in T-VEC combination therapies, other forms of treatment have also been evaluated. One example is a phase 1b clinical trial (NCT03088176) that will investigate the safety and tolerability of administering T-VEC locally, in conjunction with oral therapy with dabrafenib and trametinib. This study will be conducted with up to 20 patients with advanced melanoma who possess activating mutations in the BRAF gene. Another phase II trial (NCT02819843) intends to evaluate the effectiveness of T-VEC as a treatment for melanoma in conjunction with or without radiotherapy [42]. Interestingly, ongoing studies are investigating the potential benefits of using neoadjuvant T-VEC in patients with advanced, resectable melanoma. A phase 2 trial (NCT02211131) was conducted on 150 patients with resectable stage IIIB-IVM1a melanoma, who were randomized to receive T-VEC followed by surgery or surgery alone. The study found that the use of neoadjuvant T-VEC in combination with surgery resulted in a 25% reduction in the risk of disease recurrence compared to patients who received surgery alone [43]. Still, further research is needed to determine the best approach for utilizing T-VEC in combination with immunotherapy or other therapies for patients with advanced melanoma.

The success of T-VEC has amplified the interest of many researchers in cancer virotherapies. A number of other OVs have been designed and have undergone evaluation in preclinical and clinical studies as monotherapies or in combination with other systemic immunotherapies. For instance, TILT Biotherapeutics constructed TILT-123, an adenovirus engineered to express tumor necrosis factor (TNF)-α and IL-2. While its safety and biodistribution has been studied in mice and hamsters and it has been demonstrated to be safe in animals, the virus has been shown to induce rapid antitumor immune responses with viral replication restricted to the tumors and not normal tissues. With promising results, it is under evaluation in a phase 1 trial (NCT04217473) [44]. Another famous OV is Pexa-Vec (JX-594, Pexastimogene Devacirepvec) from SillaJen, a vaccinia virus genetically modified with thymidine kinase (TK) gene deletion and GM-CSF expression. While TK is essential for viral DNA production and has been overly expressed in the cancerous cells in comparison to in the normal cells, the deletion of the viral TK gene enables the OV to target the tumor cells more selectively while sparing the normal cells, which increases its tumor specificity. To date, JX-594 has been tested in a dozen clinical trials with many types of malignancies. All studies with JX-594 have shown excellent safety profile in more than 400 patients [45]. In general, most OVs follow the same principles regarding genetic modifications, which mainly include genetic alterations to limit pathogenicity, genomic deletions to enhance the tumor-specificity, and genomic additions to increase immune responses. While more virus species are being engineered and tested, more OVs are expected in the future to present even more options for cancer patients.

Currently, the genomic identification of cancer-promoting mutations can not only lead to drug development but can also provide information for individual patients to guide the treatment. While many drugs have shown remarkable efficacy in tumor suppression, it is still difficult to achieve a complete cure due to the refractoriness and high relapse rates of some tumors. Therefore, a combination of multiple therapies and new approaches is needed. Among the cancer therapies, the discovery of the immune checkpoints CTLA4, PD-1, and PDL1 and ICIs is a paramount achievement that has revolutionized the landscape of cancer treatment. The combination therapy of T-VEC and ICIs has, thus, appeared to be a very promising melanoma treatment approach. Indeed, many preclinical studies have provided evidence that supports the rationale of this combination. It has been shown that T-VEC can cause tumor regression and T cell infiltration, along with increased IFN-gamma and PD-L1 expression [46]. Liu et al. reported increased PD-L1 expression associated with OV monotherapy but better survival rates when combining OV with anti-PD-L1 in the mouse models [47]. The clinical trials of the combination therapy involving a variety of cancer types have also provided solid evidence for its success. At this time, there is still eager anticipation to see more trial results that may offer further insights on the future combination therapies for melanoma.

## Figures and Tables

**Table 1:** Efficacy and safety of T-VEC monotherapies and combination therapies in the treatment of skin cancers.

	Reference	Study Drugs/Mechanisms of Action	Phase (n)	Disease	Treatment	Overall Response Rate	Progression-Free Survival (Month)	Overall Survival (Month)
**I**	**T-VEC monotherapy for melanoma**							
1	A Phase I Study of OncoVEXGM-CSF, a Second-Generation Oncolytic Herpes Simplex Virus Expressing Granulocyte Macrophage Colony-Stimulating Factor [6]	talimogene laherparepvec (TVEC)/oncolytic virus therapy (OVT)	Phase I (n = 30)	Refractory cutaneous and subcutaneous metastases from breast cancer, gastrointestinal adenocarcinoma, Malignant Melanoma, and Epithelial cancer of head and neck	TVEC	N/A	N/A	N/A
2	Phase II clinical trial of a granulocyte-macrophage colony-stimulating factor-encoding, second-generation oncolytic herpesvirus in patients with unresectable metastatic melanoma [7]	TVEC/OVT	Phase II (n = 50)	Stage IIIc unresectable metastatic melanoma	TVEC	26%	N/A	16
3	Final analyses of OPTiM: a randomized phase III trial of talimogene laherparepvec versus granulocyte-macrophage colony-stimulating factor in unresectable stage III–IV melanoma (NCT00769704) [8,9]	TVEC/OVT GM-CSF/bone marrow stimulation	Phase III (n = 436)	Stage IIIB to IV melanoma	A: TVEC B: GM-CSF	31.50% 6.40%	N/A	23.3 18.9 A: 73.7% at 1 year, 49.8% at 2 year, and 38.9% at 3 year B: 69.1% at 1 year, 40.3% at 2 year, and 30.4% at 3 year
**II**	**T-VEC combinational therapy for melanoma**							
1	Randomized, open-label phase II study evaluating the efficacy and safety of talimogene laherparepvec in combination with ipilimumab versus ipilimumab alone in patients with advanced, unresectable melanoma (NCT01740297) [10]	TVEC/OVT Ipilimumab/CTLA-4 inhibitor	Phase II (n = 198)	Melanoma	A: TVEC + ipilimumab B: ipilimumab	39% 18%	8.2 6.4	86.9% at 1 year, 76.6% at 2 year 81.4% at 1 year, 67.7% at 2 year
2	A phase 1/3 multicenter trial of talimogene laherparepvec in combination with pembrolizumab for unresected, stage IIIB-IV melanoma. MASTERKEY-265 (NCT02263508) [11]	TVEC/OVT Pembrolizumab/PD-1 inhibitor	Phase 1b (n = 21)	unresectable, stage IIIB-IVM1c melanoma	A: TVEC + Pembrolizumab B: Placebo + Pembrolizumab	N/A	25.6 25.5	N/A
3	1037O MASTERKEY-265: A phase III, randomized, placebo (Pbo)-controlled study of talimogene laherparepvec (T) plus pembrolizumab (P) for unresectable stage IIIB–IVM1c melanoma (MEL). KENNOTE-034 (NCT02263508) [12]	TVEC/OVT Pembrolizumab/PD-1 inhibitor	Phase III (n = 692)	unresectable stage III-IVM1c melanoma	A: TVEC + Pembrolizumab B: Placebo + Pembrolizumab	48.60% 41.30%	14.3 8.5	66% at 2 year 49.2
4	PV-10 vs Chemotherapy or Oncolytic Viral Therapy for Treatment of Locally Advanced Cutaneous Melanoma (NCT02288897) [13]	PV-10 (10% rose Bengal disodium)/oncolytic immunotherapy	Phase III (n = 20)	Cutaneous Melanoma	A: PV-10 (10% rose Bengal disodium) B: Dacarbazine, temozolomide or TVEC	N/A Only has complete response rate (CRR)	6.1 (1.5 to 28.9) 8.6 (1.8 to 14.4)	N/A N/A
**III**	**T-VEC treatment in other cutaneous cancer types**							
1	Talimogene laherparepvec induces durable response of regionally advanced Merkel cell carcinoma in 4 consecutive patients [14]	TVEC/OVT	(n = 4)	Regionally advanced Merkel cell carcinoma	TVEC	100%	16 +	18.5 +
2	Pretreated anti-PD-1 refractory Merkel cell carcinoma successfully treated with the combination of PD-1/PD-L1 axis inhibitors and TVEC: a report of two cases [15]	TVEC/OVT	(n = 2)	Anti-PD-1 refractory Merkel cell Carcinoma	T-VEC and a PD-1/PD-L1 inhibitor	100%	N/A	N/A
3	Immunotherapy for Nonmelanoma skin cancer: Facts and Hopes (NCT02819843) [16]	TVEC/OVT	Phase II (n = 19)	Cutaneous Melanoma Merkel Cell Carcinoma Other Solid Tumors	TVEC + Radiotherapy	Study completion June 2023	Study completion June 2023	Study completion June 2023

**Table 2:** Ongoing clinical trials of T-VEC in skin cancers.

	Reference	Study Drugs/Mechanisms of Action	Stage (n)	Disease	Treatment	Key Outcomes
1	Talimogene Laherparepvec and Pembrolizumab in Treating Patients With Stage III-IV Melanoma (NCT02965716)	TVEC/OVT Pembrolizumab/PD-1 inhibitor	Phase II (n = 47)	Advanced Melanoma Refractory Melanoma	Pembrolizumab and TVEC combination	Objective response rate, median progression-free survival, median overall survival
2	T-VEC in Non-melanoma Skin Cancer (NCT03458117)	TVEC/OVT	Phase I (n = 26)	Non-melanoma Skin Cancer Basal Cell Carcinoma Squamous Cell Carcinoma Cutaneous Lymphoma Merkel Cell Carcinoma	TVEC	Local immune response, systemic immune response
3	Talimogene Laherparepvec and Nivolumab in Treating Patients with Refractory Lymphomas or Advanced or Refractory Non-melanoma Skin Cancers (NCT02978625)	TVEC/OVT Nivolumab/PD-1 inhibitor	Phase II (n = 68)	Refractory T cell Lymphoma Refractory NK cell lymphoma Cutaneous Squamous Cell Carcinoma Merkel Cell Carcinoma Other Rare Skin Tumors	TVEC followed by nivolumab and TVEC combination	Response rate, best overall response rate, progression-free survival, overall survival
4	Study of TVEC in Patients With Cutaneous Squamous Cell Cancer (NCT03714828)	TVEC/OVT	Phase II (n = 11)	Cutaneous Squamous Cell Cancer	TVEC	Overall response rate (ultrasound, targeted lesions, non-injected lesions)
5	Talimogene Laherparepvec and Panitumumab for the Treatment of Locally Advanced or Metastatic Squamous Cell Carcinoma of the Skin (NCT04163952)	TVEC/OVT Panitumumab/Anti-EGFT monoclonal antibodies	Phase I (n = 5)	Advanced Squamous Cell Cancer	Panitumumab and TVEC combination	Response rate, best overall response rate, progression-free survival, overall survival

## Data Availability

Not applicable.
